# Association of miR-608 rs4919510 polymorphism and cancer risk: a meta-analysis based on 13,664 subjects

**DOI:** 10.18632/oncotarget.9509

**Published:** 2016-05-20

**Authors:** Huiquan Liu, Yaqun Zhou, Qingquan Liu, Guangqin Xiao, Bangyan Wang, Weijuan Li, Dawei Ye, Shiying Yu

**Affiliations:** ^1^ Cancer Center, Tongji Hospital, Tongji Medical College, Huazhong University of Science and Technology, Wuhan, China; ^2^ Department of Anesthesiology, Tongji Hospital, Tongji Medical College, Huazhong University of Science and Technology, Wuhan, China; ^3^ Department of Internal Medicine, Tongji Hospital, Tongji Medical College, Huazhong University of Science and Technology, Wuhan, China; ^4^ Department of Surgery, Tongji Hospital, Tongji Medical College, Huazhong University of Science and Technology, Wuhan, China

**Keywords:** miR-608, rs4919510, polymorphism, cancer risk, meta-analysis

## Abstract

Single nucleotide polymorphisms (SNPs) in MicroRNAs (miRNAs) are involved in the mechanism of carcinogenesis. Several studies have evaluated the association of rs4919510 SNP in miR-608 with cancer susceptibility in different types of cancer, with inconclusive outcomes. To obtain a more precise estimation, we carried out this meta-analysis through systematic retrieval from the PubMed and Embase database. A total of 10 case-control studies were analyzed with 6,000 cases and 7,664 controls. The results showed that 4919510 SNP in miR-608 was significantly associated with decreased cancer risk only in recessive model (CC vs. GG+GC: OR=0.89, 95% CI: 0.82-0.97, *P*=0.009). By further stratified analysis, we found that rs4919510 SNP had some relationship with decreased cancer risk in both homozygote model (CC vs. GG: OR=0.59, 95% CI: 0.36-0.96, *P*=0.034) and dominant model (CG+ CC vs. GG: OR=0.60, 95% CI: 0.37-0.98, *P*=0.042) in Caucasians but no relationship in any genetic model in Asians. These results indicated that miR-608 rs4919510 polymorphism may contribute to the decreased cancer susceptibility and could be a promising target to forecast cancer risk for clinical practice. However, to further confirm these results, well-designed large scale case–control studies are needed in the future.

## INTRODUCTION

MicroRNAs (miRNAs) are small (usually 21–23 nucleotides in length), evolutionarily conserved, noncoding RNA molecules which participate in post-transcriptional gene regulation by binding to the complementary sequences in 3’ untranslated region (3’ UTR) of target messenger RNAs (mRNAs) [1, 2]. These miRNAs function as negative regulators through degradation of mRNAs and translational repression [3]. Accumulated evidence has suggested that the aberrations of these miRNAs are involved in cell proliferation, differentiation, migration and apoptosis in the process of carcinogenesis [4, 5]. Studies have shown that approximately 50% miRNA genes are located in cancer-related chromosomal regions [6]. The loss or gain of specific function of several miRNAs are thought to be significant events in diverse types of cancer [7]. These evidences indicated that microRNAs could be a kind of biomarkers to evaluate cancer risk.

Single nucleotide polymorphisms (SNPs), the most common genetic variation, have been demonstrated to influence the expression or target site selection of miRNAs and thus are involved in a series of biological processes by interfering interaction between miRNAs and target mRNAs [8, 9]. SNPs present in the miRNA genes have been illustrated to be a potentially important mechanism in the development and progression of cancer [10, 11].

Recently, rs4919510 SNP in miR-608 has been reported to be a predictor of clinical outcomes for patients with renal cell carcinoma [12], colorectal adenocarcinoma [13, 14], esophageal squamous cell carcinoma [15] and nasopharyngeal carcinoma [16]. However, the relationship between cancer risk and rs4919510polymorphism in miR-608 is now inconclusive and controversial. Qiu and colleagues had found that rs4919510 (C > *G*) polymorphism showed a consistent association with nasopharyngeal carcinoma susceptibility in south area of China [17]. Other study reported that there existed no significant association between miRNA-608 rs4919510 and the risk of colorectal cancer [18]. By systematically summarizing the existing data, we performed a meta-analysis to further determine whether there is an association of rs4919510 polymorphism in miR-608 with cancer susceptibility.

## RESULTS

### Characteristics of the studies

**Figure 1:**
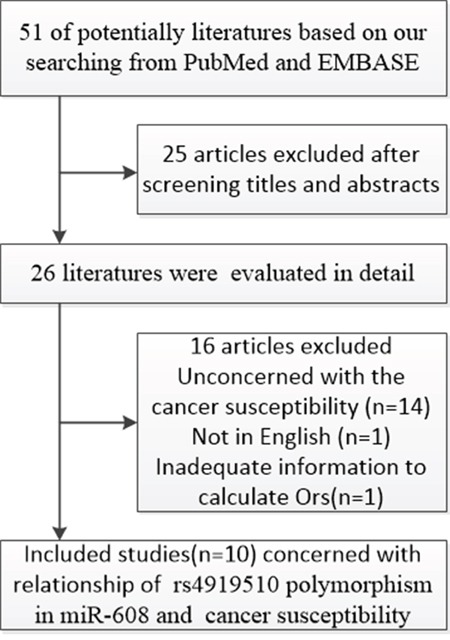
Study flow chart for the process of selecting the eligible publications

A total of 51 literatures based on our searching strategy were selected out from PubMed and EMBASE database. After screening the title and abstract, 25 studies uncorrelated with cancer risk and SNPs were excluded and 26 literatures were then evaluated in detail. Finally ten case-control studies [17–26] meeting our inclusion criteria were included into our meta-analysis with 6,000 cases and 7,664 controls (Figure 1) (Table 1). One article composed by Ryan and colleagues contains two case-control studies with detailed genotype information of Caucasian and African Americans. To perform subgroup analysis more conveniently, we regarded this one as two independent studies according to ethnicity. In all included studies, genotype distributions of rs4919510(C > *G*) in the controls were in agreement with HWE. A variety of genotyping methods were applied including Taqman, RT-PCR, MassArray, SNaPshot, and SNPstream. Genomic DNA was isolated from blood samples in all included studies except one (using both blood and tissue) [18].

**Table 1:** Characteristics of studies in the meta-analysis

Author	Year	country	Ethnicity	Cancer type	Genotyping	Source of controls	Cases(n)				Controls(n)				P value for HWE ^h^
							Total	GG	GC	CC	Total	GG	GC	CC	
Dong	2015	China	Asian	Thyroid tumor	MassArray	HB ^f^	369	136	186	47	751	279	370	102	0.494
Zhang	2015	China	Asian	ESCC ^b^	SNaPshot	PB ^g^	738	217	384	137	882	291	440	151	0.784
Yin	2015	China	Asian	Lung cancer	Taqman	HB	258	65	140	53	310	96	152	62	0.992
Wei	2015	China	Asian	Thyroid tumor	MassArray	PB	824	266	428	130	1031	326	503	202	0.950
Qiu	2015	China	Asian	SCCHN ^c^	TaqMan	PB	906	255	460	191	1072	254	532	286	0.977
Wang	2014	China	Asian	HCC ^d^	MassArray	HB	993	304	500	189	992	318	497	177	0.775
Huang	2012	China	Asian	Breast cancer	SNPstream	PB	1118	381	545	192	1417	456	684	277	0.776
Kupcinskas	2014	Lithuania	Caucasian	Gastric cancer	RT-PCR	HB	363	25	88	250	350	13	86	251	0.275
Kupcinskas	2014	Lithuania	Caucasian	CRC ^e^	RT-PCR	HB	192	7	47	138	426	12	96	318	0.364
Ryan	2012	USA	Mixed races ^a^	CRC	Taqman	PB/HB	239	19	96	124	433	36	166	231	0.729

^a^ Caucasian and African Americans; ^b^ ESCC esophageal squamous cell carcinoma; ^c^ SCCHN squamous cell carcinoma of the head and neck; ^d^ HCC hepatocellular carcinoma; ^e^ CRC colorectal cancer; ^f^ HB hospital-based; ^g^ PB population-based; ^h^ HWE Hardy-Weinberg equilibrium.

### Meta-analysis result

**Figure 2:**
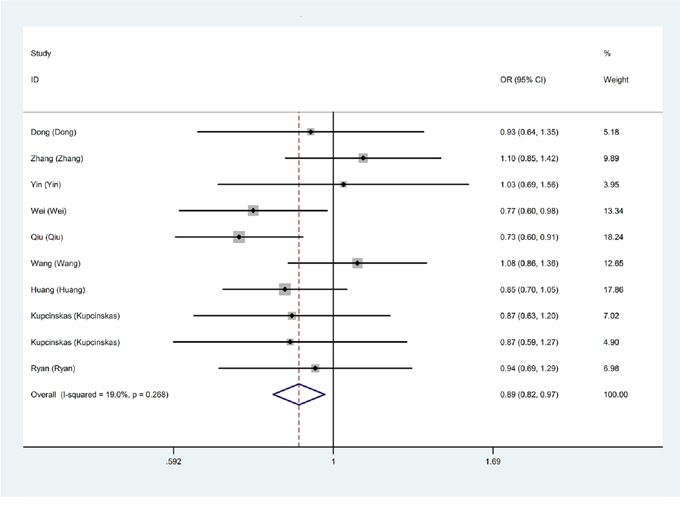
Overall meta-analysis of the relationship between miR-608 rs4919510 polymorphism and cancer risk in recessive model (CC vs. GG+GC)

The main results of this meta-analysis were shown in Table 2. For overall studies, there existed a significant association of miR-608 rs4919510 polymorphism with decreased cancer risk only in recessive model (CC vs. GG+GC: OR=0.89, 95% CI: 0.82-0.97, *P*=0.009) (Figure 2). In other genotype model, the relationship still remain controversial.

**Table 2:** The result of meta-analysis for various genotype models

		Test of Association			P Value for heterogeneity	I^2^ (%)
		OR (95%CI)^b^	Z	P Value		
Total^a^	CC vs. GG	0.90 (0.77,1.06)	1.23	0.219^c^	0.031	51.1%
	CG vs. GG	1.01 (0.93,1.10)	0.31	0.754^d^	0.361	8.8%
	CG+CC vs.GG	0.98 (0.88,1.10)	0.28	0.776^c^	0.093	39.7%
	GG+GC vs.CC	0.89 (0.82,0.97)	2.61	0.009^d^	0.268	19.0%
Asian	CC vs. GG	0.93 (0.77,1.11)	0.81	0.418^c^	0.016	61.6%
	CG vs. GG	1.02 (0.94,1.11)	0.51	0.610^d^	0.353	9.9%
	CG+CC vs.GG	1.00 (0.89,1.12)	0.01	0.988^c^	0.079	47.0%
	GG+GC vs.CC	0.90 (0.79,1.03)	1.51	0.130^c^	0.090	45.3%
Caucasian	CC vs. GG	0.59 (0.36,0.96)	2.12	0.034^d^	0.832	0.0%
	CG vs. GG	0.65 (0.39,1.09)	1.63	0.103^d^	0.727	0.0%
	CG+CC vs.GG	0.60 (0.37,0.98)	2.03	0.042^d^	0.802	0.0%
	GG+GC vs.CC	0.84 (0.68,1.04)	1.56	0.118^d^	0.876	0.0%

^a^Contrasts including homozygote model (CC vs. GG), heterozygote model (CG vs. GG), dominant model (CG+ CC vs. GG) and recessive model (CC vs. GG+GC) respectively.

^b^R odds ratio, CI confidence interval.

^c^P-value for significance under random-effects model.

^d^P-value for significance under fixed-effects model.

**Figure 3:**
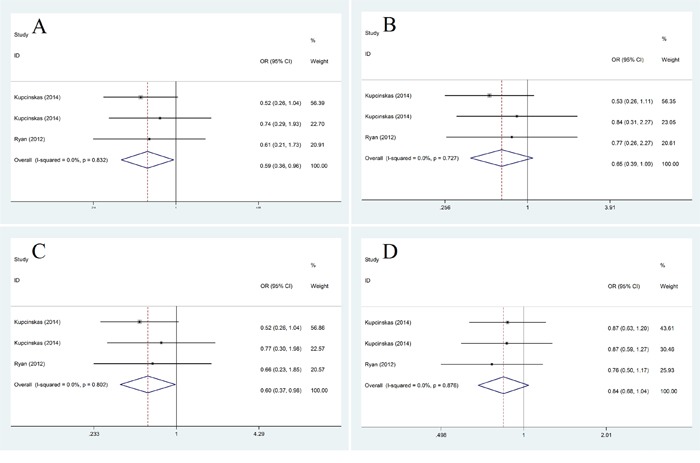
Subgroup analysis of the relationship between miR-608 rs4919510 polymorphism and cancer risk in Caucasians **A.** homozygote model (CC vs. GG); **B.** heterozygote model (CG vs. GG); **C.** dominant model (CG+ CC vs. GG); **D.** recessive model (CC vs. GG+GC).

**Figure 4:**
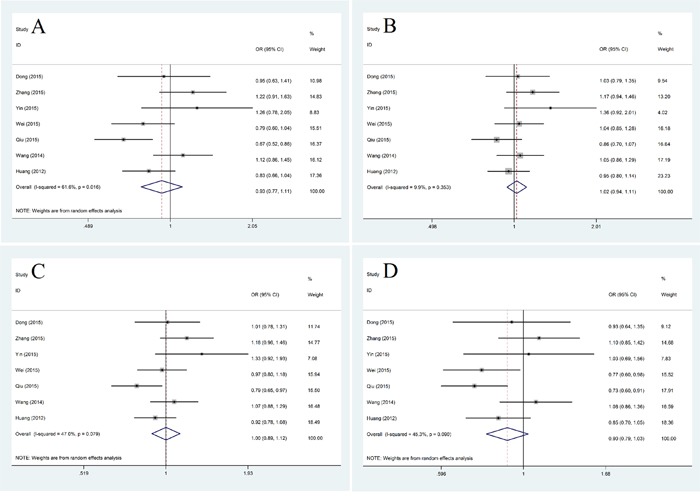
Subgroup analysis of the relationship between miR-608 rs4919510 polymorphism and cancer risk in Asians **A.** homozygote model (CC vs. GG); **B.** heterozygote model (CG vs. GG); **C.** dominant model (CG+ CC vs. GG); **D.** recessive model (CC vs. GG+GC).

For subgroup analysis of races, we found that in Caucasians miR-608 rs4919510 polymorphism had some relationship with decreased cancer risk in both homozygote model (CC vs. GG: OR=0.59, 95% CI: 0.36-0.96, *P*=0.034) and dominant model (CG+ CC vs. GG: OR=0.60, 95% CI: 0.37-0.98, *P*=0.042) (Figure 3). In Asians, Our results did not show any association of miR-608 rs4919510 polymorphism with cancer risk in any genotype model (Figure 4).

### Publication bias and sensitivity analysis

**Figure 5:**
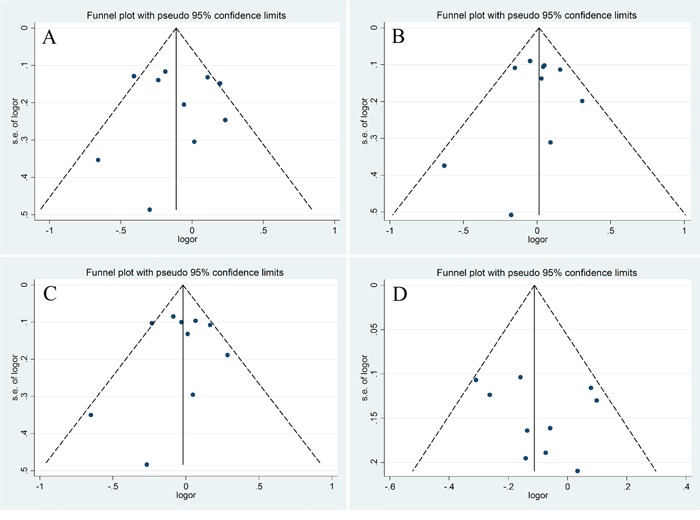
Funnel plot for publication bias test **A.** homozygote model (CC vs. GG); **B.** heterozygote model (CG vs. GG); **C.** dominant model (CG+ CC vs. GG); **D.** recessive model (CC vs. GG+GC).

**Figure 6:**
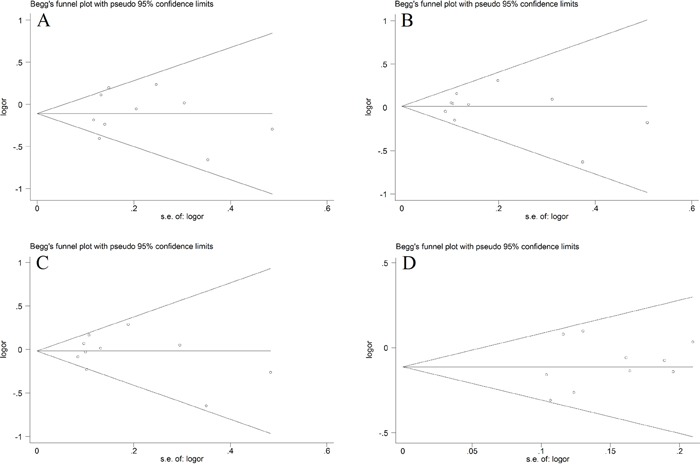
Begg's funnel plot for publication bias test **A.** homozygote model (CC vs. GG); **B.** heterozygote model (CG vs. GG); **C.** dominant model (CG+ CC vs. GG); **D.** recessive model (CC vs. GG+GC).

**Figure 7:**
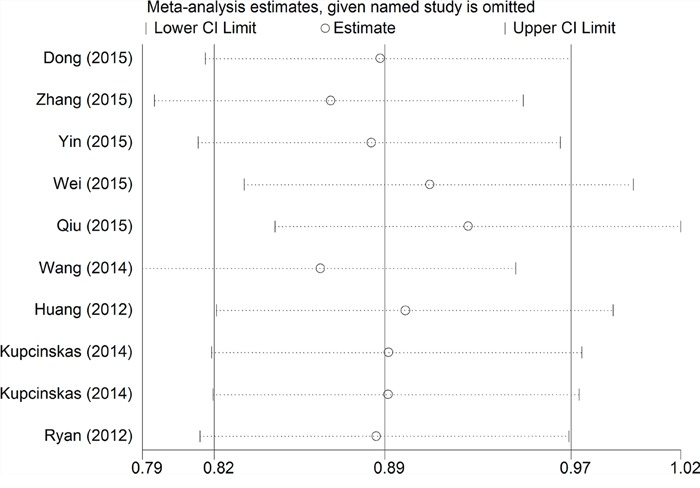
The influence of individual studies on the overall OR in recessive model (CC vs. GG+GC)

We utilized Funnel Plot, Begg's funnel plot and Egger's test to evaluate publication bias. The almost symmetrical shape of the funnel plots for four genetic models did not reveal any significant publication bias (Figure 5). There was also no evidence of publication bias in Begg's funnel for all genetic models (*P* > 0.05)(Figure 6). We also did not find out publication bias in Egger's test in homozygote model (CC vs. GG, *P*= 0.977), heterozygote model (CG vs. GG, *P*=0.744), dominant model (CG+ CC vs. GG, *P*=0.772) and recessive model (CC vs. GG+GC, *P*=0.440). Sensitivity analysis through evaluating the influence of each study on overall ORs showed that the omission of any study made no significant difference (Figure 7).

## DISCUSSION

Genetic mutations are responsible for cancer occurrence [27]. SNPs as the most common genetic sequence variation, could affect the function of a series of microRNAs by altering the formation of the primary transcript, pre-miRNA maturation, or miRNA-mRNA interactions [10, 28]. Recently, rs4919510 polymorphism in miR-608 has been reported to predict clinical outcomes for cancer patients in different cancer types [13, 14, 16]. In addition, Zhang and colleagues found that miR-608 expressions were reduced in chordoma cell lines and restoration of miR-608 inhibited chordoma cell proliferation and invasion [29]. All these studies provoked us to think about the association of miR-608 polymorphism and cancer risk.

In this meta-analysis, we demonstrated that individuals carrying CC genotype in rs4919510 have a decreased cancer risk. Moreover, Yang and colleagues reported that miR-608 suppressed the carcinogenesis of colon cancer cells *in vitro* and *in vivo* by eliminating NAA10 mRNA which participate as a key molecule in the process of carcinogenesis [30]. Taken together, these results strongly indicated that genetic variation of rs4919510 in miR-608 played an important role in cancer development.

By subgroup analysis, we found that in Caucasians miR-608 rs4919510 polymorphism had some relationship with decreased cancer risk in homozygote model and dominant model while in Asians there exist no relationship in any genotype model. These differences may be a result of various genetic backgrounds in different races and various mechanisms of carcinogenesis in different areas.

Nevertheless, some limitations in this meta-analysis should be paid attention to. First, included studies are still so limited that we cannot perform subgroup analysis for different cancer types although it is well known that one microRNA may play different functions in different types of cancer. Second, there exists a certain degree of heterogeneity between studies. After subgroup analysis stratified by races, it could be found that heterogeneity of Caucasians reduced significantly. Thus, it could be presumed that the heterogeneity partly resulted from differences in races. Simultaneously, the selection of subjects may become another source of heterogeneity. Third, only three studies were included in subgroup analysis for Caucasians and the results could be inaccurate and dubious. Fourth, only published articles were included, the unpublished and ongoing studies could convert our result. Last, we did not take sex, age, sex, family history and environmental factors into consideration and further detailed meta-analysis remain needed.

In conclusion, the results of meta-analysis indicated that rs4919510(C > *G*) polymorphism in miR-608 was significantly associated with decreased cancer risk and could become a promising target to forecast cancer risk for clinical practice. However, these results should be treated with caution due to the limitations above. For further verifying the results, well-designed large scale case–control studies are needed in the future.

## MATERIALS AND METHODS

### Publication search and data extraction

To identify all published studies concerning the relationship between miRNA polymorphisms and cancer risk, PubMed and Embase database (updated to Jan 1, 2016) were searched without language, publication, or date restrictions using the following search terms: (“microRNA 608” OR “microRNA-608” OR “miR-608” OR “rs4919510”) AND (“polymorphism” OR “SNP” OR “variation” OR “locus” OR “mutation”) AND (“cancer” OR “tumor” OR “malignance” OR “carcinoma” OR “neoplasm”). The included papers should meet criteria listed below: (1) Assessment of the relationship between miR-608 rs4919510 polymorphism and cancer risk; (2) a case-control design; (3) histologically confirmed for malignant tumors; (4) sufficient published data for further calculating odds ratios (ORs) and their 95% confidence intervals (95%CIs); (5) Meeting Hardy-Weinberg equilibrium (HWE) in the control group (P>0.05). Two reviewers (HQ Liu and BY Wang) in our group screened out the data independently and had reached a consensus on each term. All extracted data consisted of author (year), country, ethnicity, cancer type, genotyping method, source of controls, characteristics of cases and controls and P value for Hardy–Weinberg equilibrium (HWE) was exhibited in Table 1.

### Statistical analysis

We calculated P value of HWE in control group by X^2^ test and considered P value>0.05 as fulfilling Hardy-Weinberg equilibrium [31]. The association between the miR-608 rs4919510 (C > *G*) SNP and the risk of cancer was measured by odds ratios (ORs) with 95% confidence intervals (CIs) based on different genetic models such as homozygote model (CC vs. GG), heterozygote model (CG vs. GG), dominant model (CG+ CC vs. GG) and recessive model (CC vs. GG+GC) respectively. Hierarchical analysis was conducted by Ethnicity (Asian and Caucasian). The statistical significance of the pooled OR was evaluated by Z test, and P value of <0.05 was regarded as significant. Heterogeneity assumption was tested among studies using a Chi-square-based Q-test. We considered P value of > 0.10 for Q-test as a lack of heterogeneity which indicate fixed-effects model should be used to perform the meta-analysis [32]. If significant heterogeneity was existing (P<0.10 for Q-test), the random effects model should be chosen as a more appropriate one [33]. To evaluate whether there existed publication bias, Funnel plots, Begg's test and Egger's test were applied [34]. The influence of each study on overall OR was also evaluated using metaninf order. Statistical analysis was all conducted using Stata12.0 Software.
